# Between principles and practice: exploring transformative discourses in a Dutch National Park

**DOI:** 10.1007/s10531-026-03374-0

**Published:** 2026-05-21

**Authors:** Susan de Koning, Pieter Zwaan, Ingrid J. Visseren-Hamakers

**Affiliations:** 1 HAS green academy, University of Applied Sciences for Agriculture, Food, and the Living Environment, s-Hertogenbosch, The Netherlands; 2 Institute for Management Research, Department of Geography, Planning and Environment, Radboud University, Nijmegen, The Netherlands; 3 Institute for Management Research, Department of Public Administration, Radboud University, Nijmegen, The Netherlands

**Keywords:** Biodiversity, Discourses, Hollandse Duinen, Landscape, Partnership, Transformative Governance

## Abstract

Transformative change is essential to halt the worldwide decline in biodiversity. Therefore, conservation efforts must extend beyond the traditional approach of protected areas to address the complex drivers of this decline. Landscape-oriented partnerships, such as partnership-based national parks, are emerging as potential instruments for this change. To be truly transformative, we assume that such partnerships must be underpinned by a shared discourse reflecting the principles of transformative governance. Drawing on transformative change and transformative governance theory combined with Q-methodology, we have developed an approach to examining the transformative elements of partnership discourses. We applied this approach to National Park Hollandse Duinen in the Netherlands, a partnership-based national park whose vision holds transformative potential. Our study incorporated seven exploratory interviews, twenty-eight Q-methodology interviews, a workshop and participant observation in eleven meetings. Our analysis revealed four distinct partnership discourses, each of which contained transformative elements. However, none of them were fully aligned with our transformative governance framework. This finding highlights that different ideas on transformative governance do not automatically align, and that endorsing one element does not guarantee support for others. Furthermore, partners perceived tensions between transformative governance principles and the realities of partnership practice, raising questions about the potential of such partnerships to contribute to transformative change.

## Introduction

Biodiversity is declining worldwide. Currently, the International Union for Nature Conservation (IUCN, 2024) lists 46,300 species as ‘threatened’. The classical approach to nature conservation, involving protected areas and legal protection for species, has proven insufficient to halt this decline and ‘bend the curve’ of biodiversity loss (Mace et al. 2018; Leclère et al. 2020). Therefore, more profound changes are necessary. There is a growing consensus among scientists that transformative changes are needed to protect and restore biodiversity (Visseren-Hamakers et al. 2021; Pascual et al. 2022; Schmeller and Bridgewater 2023).

The Global Assessment of the Intergovernmental Science-Policy Platform on Biodiversity and Ecosystem Services (IPBES) defines transformative change as a ‘fundamental, system-wide reorganization across technological, economic and social factors, including paradigms, goals, and values’ (IPBES, 2019). More recently, the IPBES has reframed this definition to focus on ‘fundamental, system-wide shifts in views, structures, and practices’ (IPBES 2024). The views, structures and practices that require modification constitute the indirect drivers of biodiversity loss. Indirect drivers, on the other hand, are the driving forces behind direct drivers such as pollution and habitat loss (Jaureguiberry et al. 2022). Examples of indirect drivers include societal values that prioritize economic growth and thereby promote the production and consumption of unsustainable products, or trade agreements that enforce a zero-tolerance policy towards pests and thereby force farmers to use excessive amounts of pesticides.

Although many studies have conceptualized transformative change theoretically (Feola 2015; Hölscher et al. 2018; Linnér and Wibeck 2019), little research has been conducted on how to measure and achieve transformative change in practice. Although transformative changes are often defined at a societal level, for example in governance structures, laws, and regulations, they can also occur at smaller scales. Governance scholars argue that transformative changes are more likely to first take place at lower levels of governance (Torfing et al. 2024), such as the municipal or provincial level. The question therefore arises as to at what level of governance, or scale, the conservation community should focus. In recent years, landscapes have increasingly been recognized as an effective level of governance and ecological scale on which to base transformative change for biodiversity (Meijer et al. 2021; Meier et al. 2022). Landscape-oriented partnerships can be seen as a tool for governments to drive transformative change at a lower level, for example by providing funding or other incentives. These partnerships involve actors from different sectors (government, business and/or civil society) who collaborate based on shared rules, procedures, trust and social learning (Driessen et al. 2012; McNamara 2012) to develop joint objectives and strategies.

In order to contribute to transformative change for biodiversity, we propose that partners within these partnerships must share ideas about the necessity of transformative change (i.e. that the partnership should address indirect drivers) and how they can bring about such changes as a partnership (i.e. the partnership’s governance) (Newig and Fritsch 2009; Emerson and Nabatchi 2015).

In this article, we present a framework and methodology to analyze the extent to which partners of a landscape-oriented partnership share such transformative ideas. Based on existing literature on transformative change and governance, we posit that partners should support the following ideas in order to be transformative: that nature has both intrinsic and relational value (Pascual et al. 2023; IPBES 2024); that to stop biodiversity loss, we must address its indirect drivers (Díaz et al. 2019); and that we should do so through integrative, inclusive, adaptive, transdisciplinary and anticipatory governance approaches (see “Transformative ideas” section for an explanation of these governance approaches) (Visseren-Hamakers et al. 2021; Visseren-Hamakers and Kok 2022).

To analyze the extent to which these ideas are shared among the partners, we employed Q-methodology. This method analyses the ideas held by actors, how their support for specific ideas relates to their support for others, and the extent to which these ideas are shared. This set of shared ideas can be interpreted as a discourse. Q-methodology is frequently employed in the study of discourses within landscape-oriented collaborations (Langston et al. 2019, 2024; Torralba et al. 2023).

We applied this new framework and methodology to study the transformative potential of landscape-oriented partnerships in a recently established, partnership-based national park in the Netherlands: National Park Hollandse Duinen. This study builds on previous research into landscape-oriented partnerships, which revealed that the transformative intentions of such partnerships are generally unclear (de Koning et al. 2023). Our results present an in-depth analysis of the different perspectives within the park regarding its role in transformative change and governance. In our discussion, we reflect on the implications for the national park’s transformative potential, the generalizability of our findings, and their contribution to the existing literature on transformative change.

## Methods

### Case study: National Park Hollandse Duinen

**Fig. 1:**
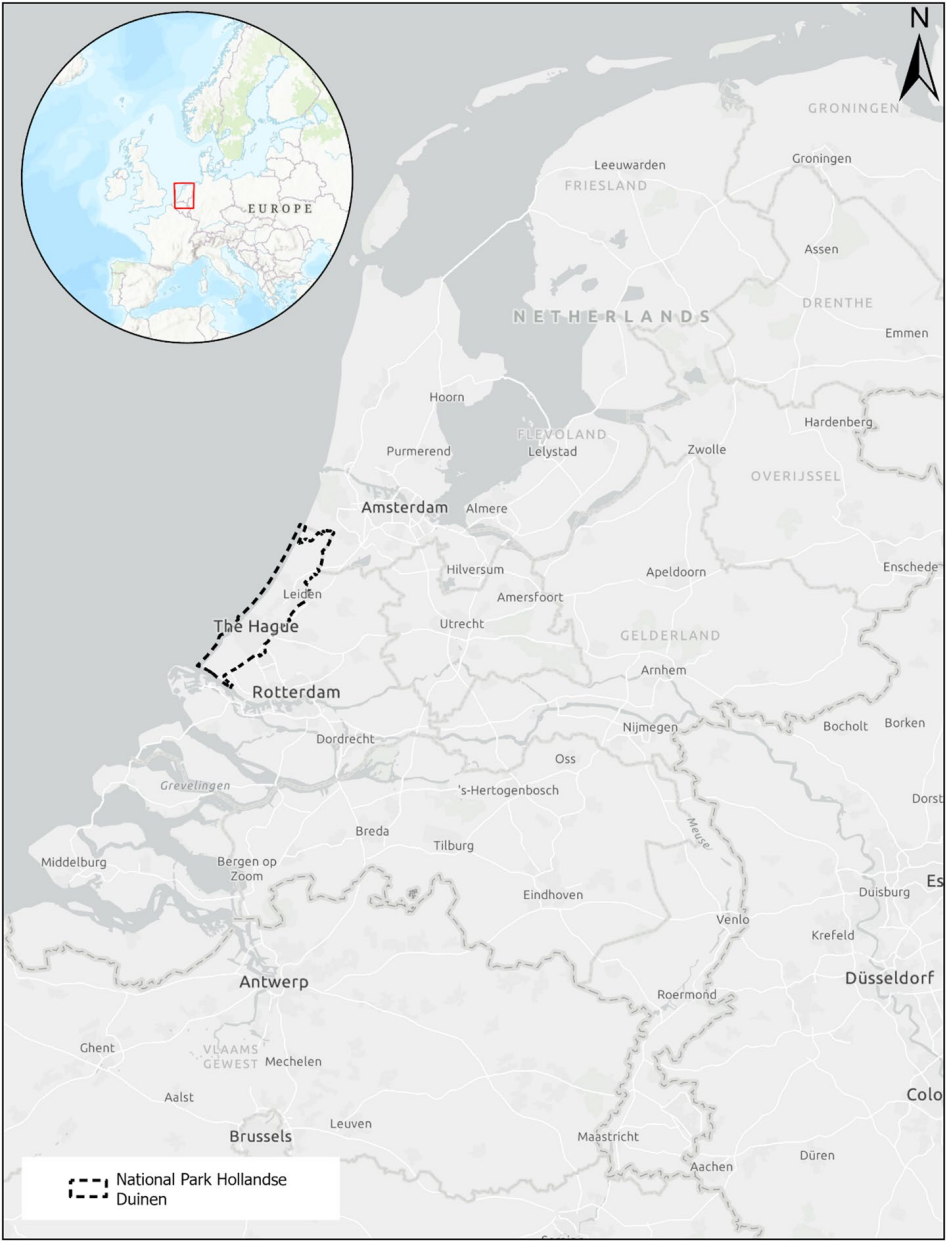
The location of National Park Hollandse Duinen in the Netherlands (map created by Hugo Langezaal)

The National Park Hollandse Duinen (NPHD) is a large, partnership-based national park located in the west of the Netherlands (see Fig. 1). It includes protected nature areas, such as Natura 2000 sites, as well as agricultural and urban areas. The park’s goal is to connect its core nature areas, which consist mainly of sandy dunes, with the surrounding landscape. These landscapes include the city of The Hague, the political heart of the Netherlands, as well as agricultural areas. The agricultural production in these areas is primarily geared towards export and includes the Westland region, which is known for its greenhouse cultivation of vegetables, and the Dutch Dune and Flower Bulb Region, where flower bulbs such as tulips and hyacinths are cultivated.

Both areas face severe sustainability challenges due to the use of pesticides and leakage of phosphate into surface water in agricultural areas, as well as a decrease in green spaces in urban areas. By incorporating these areas into the national park, NPHD aims to promote increased sustainability in the surroundings of nature areas and to enhance the natural environment. In urban areas, this could involve creating more nature-inclusive housing and buildings, for example.

The park was developed based on the concept of ‘national parks new style’. This Dutch government policy program promoted the development of national parks based on UNESCO Man and Biosphere Reserves (Commissie Verkenning Nationale Parken, 2020; NPHD 2020). The NPHD used this concept to develop a new partnership-based national park, coining the term ‘National Park 3.0’ to describe an approach that focuses not only on the geological (1.0) and biological (2.0) features of the landscape, but also on the relationship between humans and nature (3.0) (Leltz et al. 2022).

Thus, National Park 3.0 represents a new chapter in the long history of conceptualizations of national parks in the Netherlands and beyond (Janssen 2009; Kubo and Supriyanto 2010; Arpin and Cosson 2021; Kuiper et al. 2022). A common theme throughout these developments is a stronger focus on integrating nature conservation with other land uses, moving away from a land sparing approach towards a combination of land sparing and sharing (Zevenberg and van der Windt 2025). The NPHD aims to encourage land sharing, creating nature-inclusive urban and agricultural areas, as well as human-inclusive natural areas. This is intended to ensure the conservation of biodiversity in strictly protected areas and beyond. This will be achieved by collaborating with 62 partner organizations, ranging from traditional conservation bodies such as governments and nature organizations to more unconventional partners such as golf clubs and holiday parks.

We have chosen the National Park Hollandse Duinen as a case study because it is a landscape-oriented partnership with transformative potential. While the National Park’s vision does not explicitly refer to transformative change and governance, it reflects transformative ideas on the value of nature and the need to address indirect drivers. The broad partnership also allows for the incorporation of various transformative governance approaches.

### Transformative ideas

In order to understand the partners’ support for transformative change and governance, we must first establish what we mean by ‘transformative discourse’. As previously mentioned, IPBES states that transformative change encompasses changes in views, structures and practices (IPBES 2024). Drawing on academic literature on transformative change and governance, this paper focuses on the views and perspectives of actors regarding: i) the value of nature; ii) the direct or indirect drivers that should be addressed; and iii) the appropriate governance approach.

The first group of ideas consists of the values that partners assign to nature. Here, three types of values can be distinguished: (1) instrumental values, (2) relational values and (3) intrinsic values (Arias-Arévalo et al. 2017; Himes and Muraca 2018). Transformative change involves shifting the focus away from solely considering the instrumental value of nature towards recognizing the intrinsic and relational value of nature (IPBES, 2022).

The second group consists of ideas about the direct or indirect drivers that should be addressed. Direct drivers refer to the immediate causes of biodiversity loss, such as pollution or habitat fragmentation, while indirect drivers refer to the underlying causes, such as certain agricultural policies or our consumption culture (Isbell et al. 2023).

The third group consists of ideas about the governance approaches that should be used to address these drivers. According to the literature on transformative governance (Visseren-Hamakers et al. 2021; Visseren-Hamakers and Kok 2022), the following five approaches should be implemented in conjunction: integrative, inclusive, adaptive, transdisciplinary and anticipatory.

These approaches are summarized in Table 1. These approaches are summarized in Table 1.

**Table 1:** An overview of the five transformative governance approaches, based on Visseren-Hamakers et al. 2021*and* Visseren-Hamakers and Kok (2022*)*

	Governance approach	Manner of operationalization in transformative governance
Focused on addressing the indirect drivers	Integrative	Includes governance mixes Requires coordination, integration, and combination of strategies across sectors, issues, levels of governance and places
	Inclusive	Addresses power asymmetries Empowers underrepresented rights-, knowledge- and stakeholders Recognizes new and innovative rights Emancipates those representing transformative sustainability values
	Adaptive	Stimulates dialogue, learning, and reflection Reflects complexity
	Transdisciplinary	Reflects diverse values, perspectives, and knowledge systems Adopts collaborative knowledge production systems Builds capacity for transformative governance
	Anticipatory	Utilizes the precautionary principle when governing in the present for uncertain future developments, and especially the development or use of new technologies

Based on the above, we can define an ‘ideal type’ of transformative discourse as a set of shared ideas that: (1) embraces a plurality of nature-related values, including intrinsic value; (2) stresses the need to address the direct and indirect drivers of biodiversity loss; and (3) supports the use of all transformative governance approaches.

Whether partners will fully adopt this transformative discourse is an empirical question. While theory would suggest that this would be logical and consistent, in practice, partners may only share some ideas and not others, which would undermine their transformative potential.

### Q-methodology

We used Q-methodology to study the partners’ discourses on the park. This is a mixed-methods approach to studying the different shared views of a population. It enables us to examine the relationships between various themes or ideas from the participants’ perspective (Watts and Stenner 2005).

**Fig. 2:**
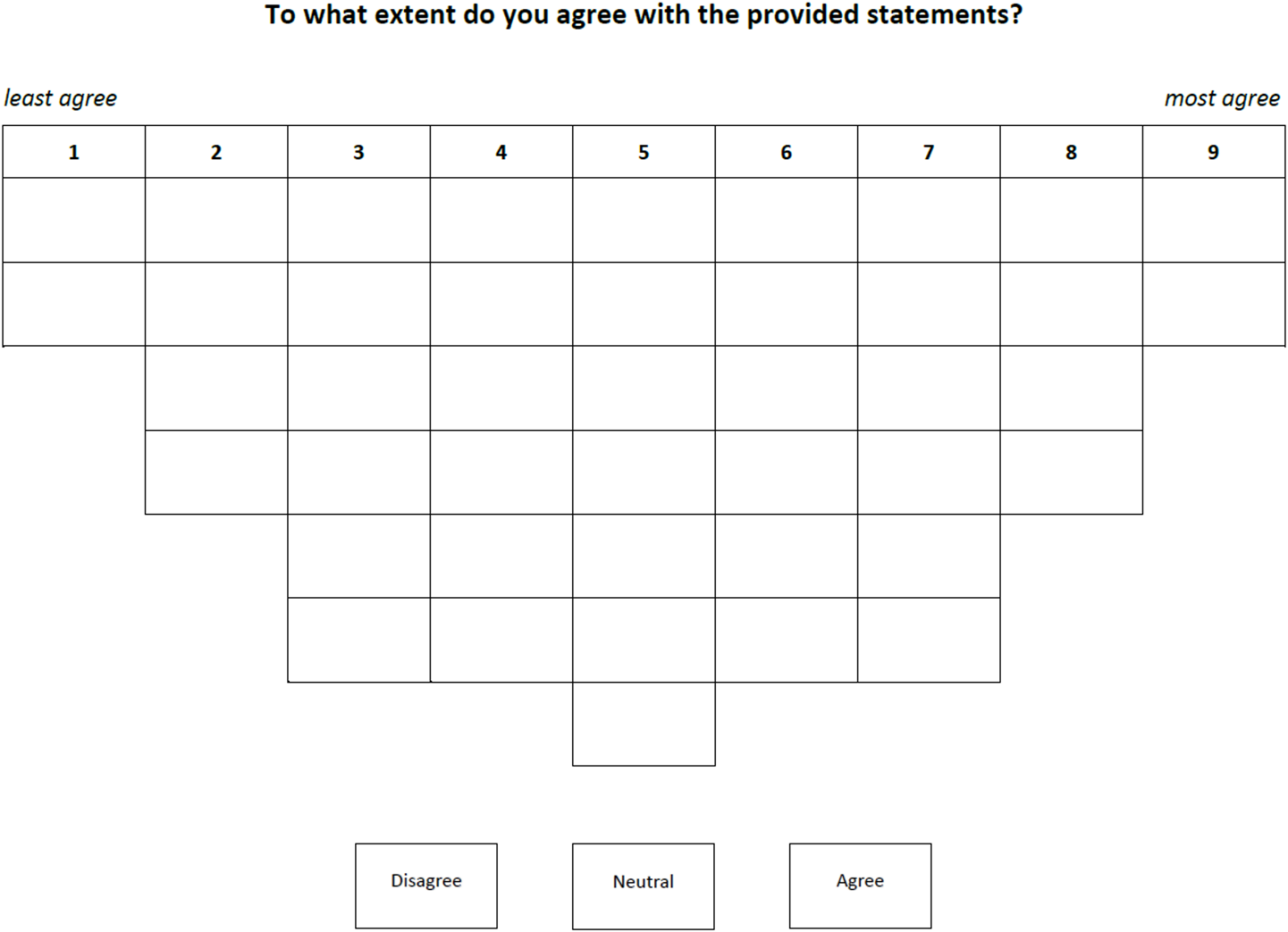
The grid used in this study (translated from Dutch to English). The grid has an inverted bell-shaped curve to force participants to make clear choices on what statements they agree most on and what statements they agree least on. Below the grid three places are created for participants to first sort the whole set of cards into three piles: disagree, neutral and agree. This first step has the function of first letting the participants get an overview of their general opinion of the cards before needing to make choices between cards in terms of which they agree most or least with

Participants are given a set of cards containing statements developed by the researchers and asked to arrange them in a grid (the Q-sort) according to their level of agreement (see Fig. 2). First, participants had to divide the cards into three piles: disagree, neutral and agree. Next, they were asked to place them within the inverted bell-curve grid. This shape of the grid forces participants to make clear choices on which statements they agreed most and which they agreed least with. The researchers then use a factor analysis to compare the different rankings of statements by the participants. The resulting factors (which indicate similar rankings) can be understood as the different discourses present in the population under study (Watts and Stenner 2005). Therefore, in the results section, we will refer to these factors as discourses.

This methodology can help us to identify areas of consensus and contrast in the perspectives of partners regarding different values, drivers and governance approaches. From a methodological point of view, the method enables us to propose and test perspectives that may not yet exist on the park by providing statements that are based on theory (Cross 2005). Furthermore, this approach can assist in testing our theoretical perspective on transformative change and governance. Q-methodology is useful for mapping coherence between different elements of a discourse (Watts and Stenner 2005), which we can use to explore relationships between transformative and non-transformative ideas.

To develop the statements, we combined transformative ideas (introduced in “Transformative ideas” section) with an exploratory study of the park and its partners. This study contextualized the transformative ideas within the park and ensured the statements were relevant and felt ‘natural’ to the respondents (McKeown and Thomas 2013).

For this study, we conducted participant observation at 11 park meetings, interviewed seven park employees and partners, and organized a workshop with five park employees (see Tables A and B in the supplementary materials). The interviews were conducted using semi-structured interview guides that focused on the park’s strengths, weaknesses and potential. During the workshop, employees were asked to deliberate on ways in which the park could contribute to transformative change and governance. The observation notes and interview transcripts were analyzed using a qualitative data analysis program called ATLAS.ti (ATLAS.ti Scientific Software Development GmbH, 2024). ATLAS.ti is a software for qualitative analysis, which can be used to create different codes that are linked to certain statements in the interviews and thereby help to structure and analyze interview data.

The statements were organized according to the type of idea (related to values, drivers, or governance) and the extent to which the idea could be regarded as transformative, based on transformative change and governance theory (see “Transformative ideas” section). Organizing the statements in this way ensured that we covered the full spectrum of ideas related to the national park.

Most statements were phrased in terms of a concrete goal or action that the park could or should have or do. In total, we developed 43 statements (see Table 2), close to the average number of statements within Q-sorts (Sneegas et al. 2021). The statements were numbered randomly to avoid influencing the participants.

**Table 2:** Statements developed for the Q-sort (translated from Dutch). Numbers behind the statements refer to the number the statement was given in the Q-sort and will be used in the remainder of the article to refer to the statement

Group of elements	More transformative	In-between	Less transformative
Values	Society should become nature-inclusive (15)	Experiencing nature should be central to the park (9)	Recreation should be central to the park (39)
	The park should contribute to a better balance between humans, nature, and the economy (37)	The park should enhance the connection between people and the landscape (33)	The park should contribute to economic development in the region (12)
Drivers	Nature cannot be protected without a transition towards sustainable agriculture (31)	Areas surrounding nature areas should be better protected (18)	Nature conservation should mainly take place within protected areas (7)
	Partner actions should contribute to changes in relevant value chains (28)	The park should inspire people to live a more sustainable lifestyle (43)	Nature conservation should not have a negative impact on export-oriented agriculture (42)
	Park developments should contribute to more sustainable policies (17)	The park should be an example for other areas where natural areas are located close to or within urban and rural areas (5)	The park should focus on concrete nature conservation or restoration projects within protected areas (30)
	The park should try to address drivers of biodiversity loss outside of the park’s borders (23)	The park should stimulate people to recreate outside of protected areas (2)	
Integrative governance	All park projects should be dealt with in an integrative manner (14)	The park should stimulate collaboration between partners (24)	The park should contribute to better coordination and alignment of activities in the park area (27)
	Nature conservation must be the point of departure for all policy processes (26)	In the park, win-win results should be strived for (11)	
Inclusive governance	Groups or organizations that prioritize nature conservation should have the strongest voice in park projects (38)	All stakeholders, including inhabitants, should be engaged in park projects (25)	Park projects should be executed by direct stakeholders (34)
	The position of groups or organizations with outspoken sustainability initiatives should be strengthened (8)	The park should give partners with ambitions for biodiversity an important say in the park (3)	Partners that are financially contributing should have the strongest voice in the decision-making processes of the park (29)
	Nature should have a voice as a stakeholder in the park (35)		
Adaptive governance	There should be active reflection on the underlying values of the park (19)	There should be active reflection on the goals of the park (22)	Nature development practices should be able to be adapted throughout the process (20)
	Continuous reflection is important to reach the goals of the park (41)		The progress of reaching park goals should be monitored (40)
Transdisciplinary governance	Inhabitants and partners should be actively engaged in developing and doing ecological research in the park (36)	Social processes, like the relationship between humans and nature, should also be studied in the park (4)	Ecological research in the park should be done by independent scientific experts (13)
	Apart from scientific knowledge, other types of knowledge and experiences should be taken into account in the development of policies (1)		
Anticipatory governance	Future generations should be taken into account in case of new developments in the park (16)	It is important to invest in taking no-regret measures within the park (21)	New developments should be given free rein in the park (32)
	Long-term thinking must become an integral part of the park’s decision-making processes (1)		It is important to take swift action (6)

After developing the statements, we created a grid ranging from − 4 to + 4 in the shape of an inverted bell curve. We presented the grid with numbers 1 to 9 to the participants. We made this choice to enable the participants to indicate where ‘disagree’ ended and ‘neutral’ or ‘agree’ started, without being steered or confused by the ‘-’ and ‘+’ symbols. We conducted a pilot study with two researchers who are not part of this author team, but who are conducting research in the National Park. During the pilot, three blank cards were given to the participants to allow them to suggest additional statements. This did not lead to any additional statements, indicating saturation. After the final alterations had been made, the Q-sort was conducted with partners of the National Park.

In total, 28 participants conducted the Q-sort. The partners (P-sample) were selected via purposeful sampling based on their role as partners (the park has different types of partners with varying levels of influence and commitment) and the type of organization (see Tables C and D in the supplementary materials). We achieved a balance between partners with more or less influence, as well as between those from different sectors (e.g. government, businesses, and civil society organizations). The park has a total of 61 partner organizations; our sample represents 23 of these.

Afterwards, participants were asked questions about their sorting in a semi-structured interview setting (see List A in the supplementary materials for all questions), the main goal of which was to facilitate the subsequent interpretation of the factors. Participants were also asked about their organization’s contribution to the park.

During the interviews, some participants were confused by statement 42 (nature conservation should not have a negative impact on export-oriented agriculture), which was phrased using the Dutch saying ‘ten koste van’. Some participants were unsure whether the statement meant that nature conservation could or should not have a negative impact on export-oriented agriculture. In cases where this statement had not been discussed during the interviews, we checked with the participants afterwards. In three cases (respondents 7, 11 and 21), the statement had been placed incorrectly and was given the opposite position in the data analysis (3 instead of 7, 3 instead of 7 and 2 instead of 8). This means that the distribution of these three Q-sorts is slightly different to that of the others. Therefore, in our analysis, we treated all sorts as having a free distribution. According to Brown (1971), there is no significant difference in factor structures when a free or forced distribution is used. Consequently, we do not anticipate that this will have had a significant impact on our results.

### Factor analysis and interpretation

We used PQmethod to analyze the Q-sorts. To determine the number of factors/discourses to retain for the final analysis, we followed Brown’s (1993) guidelines, whereby factors must have at least two significant factor loadings (± 0.48 at *p* < 0.01) from participants and the eigenvalue must exceed 1.

For factor extraction, we employed principal component analysis (PCA) with varimax rotation and automatic flagging. Using PCA with varimax rotation maximizes explained variance and prevents correlation between factors.

This led to four factors/discourses. Three out of 28 participants did not load significantly onto one of the discourses (thus not clustering around a shared view), and two out of 28 participants loaded significantly onto more than one discourse (confounded Q-sorts). We checked whether increasing or decreasing the number of discourses would decrease the number of insignificant and confounded Q-sorts, but this actually increased the number. The Q-sort data for each statement and participant can be found in Tables E and F in the appendix.

Having analyzed the factors in more detail, we analyzed the semi-structured interviews conducted after the sorting to ascertain whether the discourses were consistent with the information provided by the participants. Except for one interview in which recording was not permitted, all Q-sort interviews have been transcribed and analyzed using ATLAS.ti. In this study, we used ATLAS.ti to analyze the perspectives of participants on the specific cards. In other words, why did participants agree or not agree with a statement, and was this specific to the participant or similar among other participants sharing the same discourse.

Based on the factor analysis and the interviews, we developed narrative descriptions of the four discourses. We checked the descriptions in three ways. Firstly, we discussed our initial analysis of the discourses with employees of the park. Secondly, we wrote short descriptions of the discourses and shared these with the participants, asking for their feedback. We received feedback from ten participants (covering all four discourses), nine of whom recognized their views in the descriptions. One participant could not agree with our description, finding it too abstract. Thirdly, we discussed the discourses with a larger group of the park’s partners, including those who did not participate in the research. These partners also recognized the different discourses as being adopted and shared by partners in the park.

## Results

### Discourse analysis

Table 3 provides an overview of the results of our factor analysis of the Q-sort data. Based on this analysis, we identified four different discourses, which we labelled as follows: (1) Prioritizing nature; (2) Experiencing nature; (3) A new perspective; and (4) Spatial balance. Table 3 provides an overview of the characterizing statements (highly scored by respondents sharing a discourse) and the distinguishing statements (scored differently compared to the other discourses) for each discourse. Next, we present narrative descriptions of the four discourses, relating the statements to each other (“Four discourses” section).

“Transformative elements within the discourses” section assesses the transformative elements found in the different discourses and the extent to which consensus between the discourses can be regarded as transformative.

**Table 3:** Overview of distinguishing statements (in bold) and characteristic statements (in italics) per discourse. Some statements are both distinguishing and characteristic (italics and bold). After the statements, the Q-sort value is shown, ranging from − 4 (least agree) to + 4 (most agree)

	Discourse 1: Prioritizing nature		Discourse 2: Experiencing nature		Discourse 3: A new perspective		Discourse 4: Spatial balance	
Values	Experiencing nature should be central to the park (9)	4	The park should enhance the connection between people and the landscape (33)	4	Society should become nature-inclusive (15)	4	Experiencing nature should be central to the park (9)	3
			*Experiencing nature should be central to the park (9)*	3	*The park should contribute to a better balance between humans*,* nature*,* and the economy (37)*	3	*The park should contribute to a better balance between humans*,* nature*,* and the economy (37)*	3
			**Recreation should be central to the park (39)**	2	*The park should enhance the connection between people and the landscape (33)*	3	*The park should enhance the connection between people and the landscape (33)*	3
					***Experiencing nature should be central to the park (9)***	-2	**The park should contribute to economic development in the region (12)**	2
					***Recreation should be central to the park (39)***	-3		
Drivers	***Areas surrounding nature areas should be better protected (18)***	4	**Nature cannot be protected without a transition towards sustainable agriculture (31)**	-1	*The park should try to address drivers of biodiversity loss outside of the park’s borders (23*	3	**The park should stimulate people to recreate outside of protected areas (2)**	2
	***Nature cannot be protected without a transition towards sustainable agriculture (31)***	3	**Areas surrounding nature areas should be better protected (18)**	-2	*Nature conservation should mainly take place within protected areas (7)*	-3	*Nature conservation should not have a negative impact on export-oriented agriculture (42)*	-2
	*The park should try to address drivers of biodiversity loss outside of the park’s borders (23)*	3	**Nature conservation should mainly take place within protected areas (7)**	-3	*Nature conservation should not have a negative impact on export-oriented agriculture (42)*	-4	***The park should focus on concrete nature conservation or restoration projects within protected areas (30)***	-3
	**The park should focus on concrete nature conservation or restoration projects within protected areas (30)**	2	***The park should inspire people to live a more sustainable lifestyle (43)***	-4			*Nature conservation should mainly take place within protected areas (7)*	-4
	*Nature conservation should take mainly place within protected areas (7)*	-3						
	*Nature conservation should not have a negative impact on export-oriented agriculture (42)*	-4						
Integrative governance	**The park should stimulate collaboration between partners (24)**	-1	*In the park*,* win-win results should be strived for (11)*	3	***All park projects should be dealt with in an integrative manner (14)***	2	***The park should stimulate collaboration between partners (24)***	4
	*The park should contribute to better coordination and alignment of activities in the park area (27)*	-3	***The park should stimulate collaboration between partners (24)***	3	**In the park**,** win-win results should be strived for (11)**	1	***All park projects should be dealt with in an integrative manner (14)***	4
	***In the park***,*** win-win results should be strived for (11)***	-3	**Nature conservation must be the point of departure for all policy processes (26)**	-4	**The park should stimulate collaboration between partners (24)**	0	*In the park*,* win-win results should be strived for (11)*	3
							*The park should contribute to better coordination and alignment of activities in the park area (27)*	2
Inclusive governance	***Nature should have a voice as a stakeholder in the park (35)***	3	***All stakeholders***,*** including inhabitants***,*** should be engaged in park projects (25)***	4	**All stakeholders**,** including inhabitants**,** should be engaged in park projects (25)**	1	**Park projects should be executed by direct stakeholders (34)**	1
	**The park should give partners with ambitions for biodiversity an important say in the park (3)**	2	**Nature should have a voice as a stakeholder in the park (35)**	1	*Partners that are financially contributing should have the strongest voice in the decision-making processes of the park (29)*	-2	**All stakeholders**,** including inhabitants**,** should be engaged in park projects (25)**	0
	**Groups or organizations that prioritize nature conservation should have the strongest voice in park projects (38)**	1	**Partners that are financially contributing should have the strongest voice in the decision-making processes of the park (29)**	0	***Park projects should be executed by direct stakeholders (34)***	-3	*Groups or organizations that prioritize nature conservation should have the strongest voice in park projects (38)*	-3
	**All stakeholders**,** including inhabitants**,** should be engaged in park projects (25)**	-2	*The park should give partners with ambitions for biodiversity an important say in the park (3)*	-2	*Groups or organizations that prioritize nature conservation should have the strongest voice in park projects (38)*	-3	*The position of groups or organizations with outspoken sustainability initiatives should be strengthened (8)*	-3
	*Partners that are financially contributing should have the strongest voice in the decision-making processes of the park (29)*	-2	*The position of groups or organizations with outspoken sustainability initiatives should be strengthened (8)*	-3				
			*Groups or organizations that prioritize nature conservation should have the strongest voice in park projects (38)*	-3				
Adaptive governance			*The progress of reaching park goals should be monitored (40)*	2	*The progress of reaching park goals should be monitored (40)*	3		
			**Continuous reflection is important to reach the goals of the park (41)**	2				
Transdisciplinary governance	**Social processes**,** like the relationship between humans and nature**,** should also be studied in the park (4)**	-1	*Apart from scientific knowledge*,* other types of knowledge and experiences should be taken into account in the development of policies (1)*	2			***Social processes***,*** like the relationship between humans and nature***,*** should also be studied in the park (4)***	-3
	**Apart from scientific knowledge**,** other types of knowledge and experiences should be taken into account in the development of policies (1)**	-2						
Anticipatory governance	*It is important to take swift action (6)*	-3	*Future generations should be taken into account in case of new developments in the park (16)*	3	*Future generations should be taken into account in case of new developments in the park (16)*	4	*New developments should be given free rein in the park (32)*	-4
	*New developments should be given free rein in the park (32)*	-4	**It is important to invest in taking no-regret measures within the park (21)**	1	**It is important to take swift action (6)**	2		
			*It is important to take swift action (6)*	-3	*New developments should be given free rein in the park (32)*	-4		
			*New developments should be given free rein in the park (32)*	-3				

### Four discourses

The factor analysis revealed four distinct discourses on the park. The first, which we termed ‘Prioritizing Nature’, emphasizes the protection of nature for its own sake and for people’s enjoyment, as well as advocating conservation both inside and outside designated areas. This discourse prioritizes biodiversity over broad inclusiveness, giving more weight to actors with strong environmental ambitions. It recognizes indirect drivers, such as the export orientation of agriculture, but pays little attention to adaptive or anticipatory governance.

The second discourse, ‘Experiencing nature’, centers on recreation and nature experiences. It frames the park as a collaborative arrangement that balances the landscape’s multiple functions. Here, nature is one of many stakeholders with an equal voice. Inclusivity, reflection and monitoring are valued, but biodiversity conservation and the drivers of biodiversity loss receive less focus.

The third discourse seeks to integrate nature more fully into society by balancing ecological and economic considerations, and by fostering public awareness and action. We therefore call this discourse ‘A new perspective’. Conservation should extend beyond official reserves, with immediate and long-term measures in place. It strongly supports anticipatory governance, transformative values and addressing indirect drivers.

The final discourse highlights the need for spatial balance between economic development and nature experiences. While it does not emphasize concrete conservation projects, it promotes recreation in less sensitive areas to reduce pressure on protected sites. Although collaboration and integrated approaches are central, less attention is given to nature inclusivity, adaptive governance or the drivers of biodiversity loss.

### Transformative elements within the discourses

By examining how participants adopting a specific discourse ‘score’ the different statements, we can analyze the extent to which the discourses incorporate transformative ideas, as well as the elements (e.g. values or drivers) of transformative change and governance that are considered important in the context of the park.

**Fig. 3:**
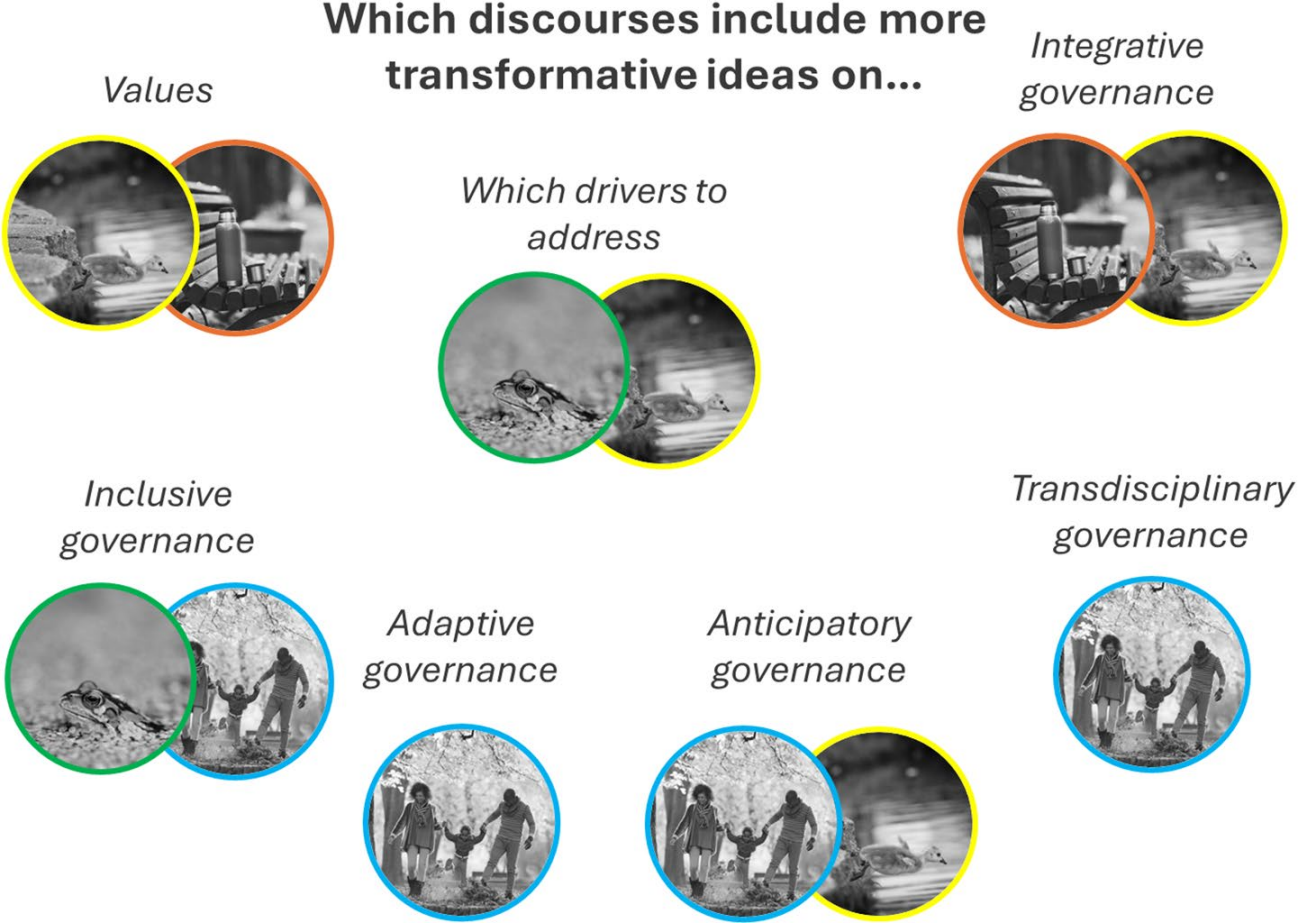
An overview of which discourses have at least one positively scored ( > + 1) characterizing statement that is labelled as more transformative. Green=Discourse 1: Prioritizing nature; Blue=Discourse 2: Experiencing nature; Yellow=Discourse 3: A new perspective; and Brown=Discourse 4: Spatial balance

Figure 3 provides a visual summary of which transformative elements feature in which discourses. This figure shows that, while all transformative statements received support from partners, this was not the case for all discourses or at the same time. For instance, discourse 1, ‘Prioritizing nature’ (in green), includes more transformative statements regarding drivers and inclusive governance, whereas discourse 3, ‘Spatial balance’ (in brown), features more transformative statements relating to the values of nature and integrative governance. Interestingly, discourse 2, ‘Experiencing nature’ (in blue), supports several transformative governance approaches but does not explicitly support transformative values regarding nature or recognize the park’s role in addressing indirect drivers. In contrast, discourse 3, ‘A new perspective’ (in yellow), is the only discourse that explicitly supports transformative statements relating to both values and drivers. According to transformative change literature (Visseren-Hamakers and Kok 2022), this is seen as a prerequisite for being transformative.

Examining the statements that were scored similarly across the discourses (see Table F in the supplementary materials), we observe that these statements tend to be scored neutrally across all discourses. In other words, the discourses are similar in that they do not score any statements either negatively or positively. For instance, the notion that partners’ actions should impact relevant value chains and that park developments should promote sustainable policies was neither explicitly endorsed nor rejected. During the interviews, it became clear that most partners primarily view the park as a tool for nature conservation rather than for increasing sustainability more generally. While partners differ in their views on the potential for economic development in the landscape, they do agree that new developments should never be given free rein, reflecting a shared stance against uncontrolled change in conservation and spatial planning. However, the fact that most partners focus on direct rather than indirect drivers of biodiversity loss could seriously inhibit the park’s transformative potential.

Another shared idea is that actively reflecting on values and involving inhabitants or partners in research is not important for achieving the park’s goals. Instead, partners seem to agree that the park is about experiencing nature and connecting with the landscape and that further reflection is unnecessary, as concrete action is needed instead. While the landscape focus brings all actors together, without active reflection on what the landscape entails and how this focus can help protect biodiversity, its transformative potential may be limited.

## Discussion

This research started from the assumption within transformative governance theory that to realize transformative change, it is crucial to implement all transformative governance approaches concurrently while focusing on indirect drivers and different values of nature (Visseren-Hamakers et al. 2021; Visseren-Hamakers and Kok 2022). The framework presented in this study allowed us to evaluate the extent to which partners in a landscape-oriented partnership recognize and share the importance of these approaches.

Using our framework and methodology, we gained a detailed understanding of the level of support that partners have for these ideas in practice. While the specific statements, and thus the discourses that followed from these, are specific to the partnership under study, our framework and methodology can be applied to other landscape governance processes to map stakeholders’ views on transformative change and governance. To explore whether we see similar trends in terms of types of discourses (e.g. prioritizing versus inclusive discourses), there is a need for further research, for example by applying Q-methodology to a larger number of partnerships and partners, or to combine it with a survey. The developed statements and identified discourses can form a basis for this work.

Below, we discuss three important reflections for broader literature and research on transformative governance based on this study: (1) tensions between different transformative governance approaches; (2) the need for and possibility of developing a shared transformative discourse; and, related to that, (3) the implications for the transformative potential of landscape-oriented partnerships.

Examining how participants scored statements related to transformative change and governance revealed that all discourses featured transformative ideas to some extent. This aligns with the expectation that landscape-oriented governance provides a potential site for transformative change. However, none of the discourses embraced all types of transformative ideas, such as with regard to values, issues to address, and governance approaches. Therefore, our findings show that the discourses differ in terms of which elements of transformative change and governance they support. These findings are consistent with a recent systematic analysis of landscape-oriented partnerships worldwide (de Koning et al. 2023), which revealed that most partnerships adopt only certain elements of a transformative governance approach, primarily integrative and inclusive governance. In practice, therefore, transformative governance theory seems to be only partially supported.

This study enabled us to investigate this selective support further. Based on interviews conducted after the statements were sorted, we found that one explanation for this is the *perceived* contradiction between several transformative governance principles. Several participants indicated that they experience tension between different elements within the framework, as well as between these theoretical principles and the practical reality of a landscape-oriented partnership.

We found that partners aiming to balance the different functions and spatial claims of the park (adopting discourse 2, ‘Experiencing nature’ or discourse 4, ‘Spatial balance’) experience this tension particularly. These partners support integrative, inclusive, adaptive, anticipatory, and transdisciplinary governance, but feel that it is not possible to start a partnership with strict set objectives or principles - let alone prioritize a certain view of, or function for, the park - due to this perceived contradiction. Apart from partners supporting discourse 1 (‘Prioritizing nature’), all other partners feel that the park’s goal is to connect partners with different interests, meaning it should not prioritize or emancipate specific actors or interests.

In other words, partners feel that the park should be inclusive, but not necessarily in a strong, transformative, or emancipatory way. They view this open, pluralistic inclusivity as an integral component of the landscape-oriented partnership approach, in which all types of partners (and interests) connected by a shared landscape collaborate. The finding is strengthened by the outcomes of a Finnish case study on transformative governance in the plastics sector. Here, similar tensions are also recognized by the partners. The authors of the study argue that it is difficult to be fully transformative in a collaborative setting as transformative change will inevitably harm existing unsustainable interests (Sundqvist and Åkerman 2024) which could prevent these partners from participating in the partnership.

Drawing on literature concerning collaborative governance (Newig and Fritsch 2009; Ansell and Gash 2008), we assumed that adopting a transformative discourse from the outset, or developing one through collaborative governance, is essential for achieving transformative change. From this perspective, it seems problematic that partners support different discourses that are all not fully transformative. Moreover, the possibility of moving towards a shared transformative discourse also seems limited for the following reasons. A shared discourse would probably be based on the so-called consensus statements. Consensus statements are the statements that were scored similarly among the different discourses. Based on the consensus statements, we see that, firstly, and unsurprisingly, such a discourse would focus strongly on the landscape. All partners wish to protect and experience the landscape. This aligns with the primary rationale for the effectiveness of landscape-oriented partnerships (Meijer et al. 2021). While this binds all partners together, this basis may be too limited to further develop shared ideas on transformative change. Indeed, this focus on the landscape appears to translate into a focus on ‘concrete’ biodiversity and nature, such as species conservation and nature restoration within the park boundaries. The other consensus statements mainly show a wide array of topics that all partners adopt a neutral stance towards. All these ‘neutral’ topics are related to addressing indirect drivers related to broader sustainability, such as influencing policies or value chains as a partnership. Thus, the fact that these statements are scored neutral among the different discourses shows that most partners wish to remain ‘apolitical’ instead of addressing the indirect drivers of biodiversity loss.

If these shared ideas were to form the basis of a compromise, partners supporting transformative approaches would need to adopt a less transformative stance. Searching for a compromise would thereby reduce the transformative potential and ambitions of most partners (Dupuis et al. 2023). While most partners support the current vision of the park (NPHD 2020), some partners also criticized the ‘something for all’ approach in the interviews, as this prevents the allocation of park resources towards a clearly defined transformative goal.

This need for a shared vision was further questioned during a plenary reflection on our research results with the park’s partners. The diversity of discourses about the park was welcomed, based on the idea that different partners could play different roles depending on which transformative ideas they support. Existing literature on collaborative governance suggests that this could work for partnerships as long as there is ‘mutual understanding’ of each other’s views and interests (e.g. Emerson and Nabatchi 2015). Based on this mutual understanding, policies that go beyond a basic compromise may be developed and negotiated (Forester 2006). While this *negotiated view* can increase the transformative potential of the partnership compared to a *shared compromise*, it remains to be seen whether this negotiated outcome will result in sufficient transformative change.

Additional longitudinal research is therefore needed to determine how different discourses develop over time, and the impact this has on the potential for transformative change.

Thus, the question remains of what these findings tell us about the contribution of landscape-partnerships to transformative change. While landscape-oriented partnerships are considered promising policy venues to realize this, our study shows that the inclusiveness of partnerships can hinder transformative change. Without addressing the underlying power relations between partners, landscape-oriented partnerships risk perpetuating the status quo rather than facilitating transformative change. The transformative potential of partnerships will therefore be dependent on their capacity to strengthen the position of partners that support transformative ideas, to be selective in the type of organizations that are engaged as partners in the development of the partnership, and/or the potential to develop a negotiated view that holds all the different transformative approaches that are supported among the partners.

## Data Availability

No datasets were generated or analysed during the current study.
